# Denosumab-Induced Hypocalcemia after Billroth II Gastric Bypass Surgery

**DOI:** 10.1155/2020/8833723

**Published:** 2020-07-23

**Authors:** Abigail M. Schmucker, Dina E. Green, Philip M. Montemuro

**Affiliations:** ^1^Sidney Kimmel Medical College, Thomas Jefferson University, 1025 Walnut Street, Philadelphia, PA 19107, USA; ^2^Lankenau Medical Center, 100 East Lancaster Avenue, Wynnewood, PA 19096, USA

## Abstract

Hypocalcemia is a known risk following bariatric surgery and can contribute to the development of osteoporosis. Osteoporosis is commonly treated with denosumab, though denosumab can exacerbate underlying abnormalities in calcium homeostasis. We present the case of a 59-year-old female with severe hypocalcemia who had been treated with denosumab for osteoporosis three months before and had Billroth II gastric bypass surgery 15 years before, for bariatric purposes. Intravenous calcium supplementation was used to correct the initial electrolyte abnormality, and the patient was able to maintain appropriate calcium levels on high doses of oral calcium before discharge. Denosumab-induced hypocalcemia has been previously reported in patients with predisposing conditions including chronic kidney disease, primary sclerosing cholangitis, Crohn's disease, and a history of sleeve gastrectomy for marginal gastric ulcers. A few cases of hypocalcemia have been reported in patients with a history of bariatric surgery secondary to vitamin D deficiency, but this report is unique in demonstrating denosumab-induced hypocalcemia after bariatric surgery with normal vitamin D levels, suggesting a primary malabsorption of calcium. The risk of severe hypocalcemia should be considered before initiating denosumab to treat osteoporosis in patients with a history of bariatric surgery. If denosumab is initiated, serum calcium levels should be closely monitored, and patients should be educated about the importance of adherence to calcium supplementation.

## 1. Introduction

With rising rates of obesity globally, bariatric surgery for weight loss has become an increasingly safe and effective option for managing excess weight and associated metabolic and mechanical complications [1, 2]. While bariatric surgical procedures continue to be modified to maximize weight loss while minimizing morbidity and mortality, they are not without risk [1]. Suboptimal preoperative nutrition, anatomical changes, and postoperative dietary constraints can lead to significant micronutrient and vitamin deficiencies after bariatric surgery, including hypocalcemia [2].

The combination of these nutritional deficiencies with mechanical and hormonal changes resulting from bariatric surgery can also contribute to the development of osteoporosis [3]. Antiresorptive therapies such as bisphosphonates and denosumab are first-line treatments for osteoporosis, yet they also pose a risk of hypocalcemia in predisposed patients, ranging from minor and asymptomatic to life-threatening conditions [4, 5]. Hypocalcemia is a known risk of both bariatric surgical procedures and antiresorptive osteoporosis therapies [2, 4, 6], but the hypocalcemia risk of these two factors combined and the optimal treatment of those with osteoporosis secondary to a history of bariatric surgery have received little attention in the literature.

In this report, we present the case of a 59-year-old female who developed severe hypocalcemia three months after treatment with denosumab for osteoporosis and fifteen years after Billroth II gastric bypass surgery. This case was prepared in accordance with the CARE guidelines for case reports (see Appendix 1 in Supplementary Materials for CARE checklist).

## 2. Case Presentation

A 59-year-old postmenopausal woman with a past medical history of Billroth II gastric bypass surgery 15 years before for bariatric purposes, Barrett esophagus, pericarditis secondary to suspected rheumatologic disease status treated with a pericardial window 3 years before, rheumatoid arthritis with negative rheumatoid factor, paroxysmal atrial fibrillation, amiodarone-induced hypothyroidism, and osteoporosis treated with denosumab 3 months before was referred to the emergency department by her endocrinologist after outpatient labs revealed profound hypocalcemia, hypophosphatemia, and hypomagnesemia. The patient had recently returned from vacation, where she experienced 2-3 episodes of diarrhea. She reported compliance to her medications, which included 50,000 IU ergocalciferol daily and 630 mg calcium citrate 2-3 times daily, apart from taking a decreased dose of calcium for the last 4 days of her vacation since she was running low on pills. She noted fatigue and intermittent tingling in her hands, feet, and perioral region for 2-3 days prior to admission. She denied weight loss, fever, muscle cramping, seizures, loss of consciousness, or lightheadedness.

On presentation, the patient was afebrile with a temperature of 98.2ºF, blood pressure of 136/74 mmHg, heart rate of 71 beats/min, and respiratory rate of 15 breaths/min. Her oxygen saturation was 100% on room air. Physical examination revealed a well-appearing woman with a body mass index (BMI) of 30 kg/m^2^ in no acute distress. Neck was supple with no thyromegaly. There were no abnormalities on cardiac or pulmonary exam. Abdomen was soft, nontender, and nondistended with normal bowel sounds. Patient had a positive Chvostek's sign. No muscle twitches or spasms were appreciated. Strength, sensation, reflexes, and cranial nerves were intact. The patient had no signs of confusion or psychosis.

Blood tests from the emergency department revealed a calcium of 5.8 mg/dL, phosphorus of <1 mg/dL, and magnesium of 1.7 mg/dL. Albumin was 3.8 g/dL, so the corrected calcium was 6.0 mg/dL. Ionized calcium was 0.8 mg/dL. Parathyroid hormone was 136 ng/L. 25-Hydroxy vitamin D was normal at 46 ng/mL, and 1-25-hydroxy vitamin D was normal at 30 pg/mL. Urinalysis was within normal limits, and urine calcium was <2 mg/dL. Creatinine was 0.61 mg/dL, and blood urea nitrogen was 11 mg/dL. TSH was normal at 0.78 *μ*U/mL. An electrocardiogram performed in the emergency department showed normal sinus rhythm with a corrected QT interval of 493 ms.

In the emergency department, the patient was given 2 g of IV magnesium sulfate, 30 mmol of IV sodium phosphate, and 1 g of IV calcium gluconate. During her 4-day hospital stay, the patient continued to receive IV calcium, as well as oral calcitriol, phosphate, and magnesium. When she was able to consistently maintain serum calcium of at least 8.0 mg/dL on oral calcium citrate supplementation, she was discharged with a calcium level of 8.0 mg/dL, magnesium of 1.9 mg/dL, and phosphorus of 2.3 mg/dL. She was instructed to take calcium citrate of 1260 mg 4 times daily, calcitriol of 0.25 mcg 2 times daily, phosphorus of 250 mg 3 times daily, and magnesium oxide of 400 mg 2 times daily and to be followed up closely with her endocrinologist. Since discharge, the patient has been able to maintain adequate calcium levels on a low dose of calcium supplementation, and she is interested in considering further doses of denosumab.

## 3. Discussion

The risk of hypocalcemia following denosumab use for osteoporosis has been demonstrated in multiple case reports and case series for patients with chronic kidney disease [7–10] and in individual case reports for patients with primary sclerosing cholangitis [11], Crohn's disease [8, 12, 13], and a history of sleeve gastrectomy for marginal gastric ulcers [12]. A few cases of hypocalcemia with denosumab have been reported in patients with a history of bariatric surgery, but each of these was likely secondary to vitamin D deficiency [14, 15]. In our case, normal active and storage forms of vitamin D, low phosphate, and normal kidney function tests suggested that the hypocalcemia was not due to renal failure or vitamin D deficiency. With these etiologies excluded, the most likely cause of hypocalcemia was primary calcium malabsorption from the bariatric Billroth II procedure 15 years before, exacerbated by denosumab use 3 months before and insufficient calcium supplementation during the week prior to admission.

Billroth II gastrectomy is a form of gastric bypass surgery often used for refractory peptic ulcer disease and gastric adenocarcinoma, but it can also be used for weight loss since it reduces the volume of the stomach, causing early satiety [16]. Due to a combination of anatomical changes, postoperative dietary changes, and suboptimal preoperative nutrition, many patients develop micronutrient deficiencies after bariatric surgery [2], and Billroth II procedure has been shown to cause calcium and vitamin D absorption disturbances [17]. Because of the risk of hypocalcemia following bariatric surgery, calcium supplementation and vitamin D supplementation are recommended to prevent postoperative hypocalcemia [14].

Even with adequate supplementation, the malabsorption resulting from bariatric surgery, combined with mechanical and hormonal factors related to weight loss, predisposes patients to osteoporosis after bariatric surgery [3]. Denosumab, a common first-line treatment for osteoporosis, is a monoclonal antibody approved for prevention of skeletal-related events in postmenopausal women at risk for osteoporosis (as Prolia®) and is given as a subcutaneous injection every six months [4, 18]. Denosumab functions by inhibiting osteoclast-mediated bone resorption by binding to the receptor activator of nuclear factor kappa B ligand (RANKL), thus preventing RANKL from binding to its receptor. As a result, less skeletal calcium is released into the circulation [19]. Denosumab is known to exacerbate existing hypocalcemia, so correction of preexisting hypocalcemia is suggested prior to administration. Additionally, monitoring of calcium, phosphorus, and magnesium is recommended, and supplementation with at least 1000 mg daily of calcium and 400 IU of vitamin D is recommended, particularly during the first 14 days after administration [20].

Our patient experienced profound hypocalcemia 15 years after bariatric surgery and 3 months after denosumab administration. Prior to this episode, the patient was on a longstanding dose of 50,000 IU ergocalciferol daily, as well as 630 mg calcium citrate 2-3 times daily, which is well within the recommendations of the Prolia® insert [20]. She was closely monitored by her rheumatologist and endocrinologist during the interval immediately after denosumab administration, and she never experienced profound hypocalcemia during this time. Considering our patient had two separate indications for calcium and vitamin D supplementation, perhaps she needed higher doses of oral supplements, or she was particularly susceptible to a few days of a decreased calcium dose while on vacation.

One previous study sought to identify risks for denosumab-induced hypocalcemia and found no significant associations between clinical parameters and development of hypocalcemia [5]. Notably, however, previous gastrointestinal surgeries were not included among the clinical parameters they assessed. The study concluded that high baseline bone turnover, as evidenced by increased serum bone turnover markers, was associated with denosumab-induced hypocalcemia [5]. Given that our patient likely had a decreased ability to absorb calcium via the gastrointestinal tract, it seems reasonable to hypothesize that she had high baseline bone turnover.

Strengths of this case include the fact that the patient was a reliable historian with a well-documented surgical and medical history within the electronic medical record. Additionally, her established outpatient endocrinologist followed her in the hospital, allowing for continuity of care and effective outpatient follow-up. A weakness of the case is that there is no way to parse out the relative contribution to hypocalcemia of each potential factor—denosumab use, chronic absorption issues from prior bariatric surgery, and missed calcium supplementation and diarrhea in the days leading up to admission. Additionally, given the timing of the patient's bariatric surgery relative to the current episode, we do not have access to the degree of her weight loss after the surgery; however, based on the history from the patient and a current BMI of 30, we do not suspect that malabsorptive issues were a result of an overly aggressive bariatric surgery. Further research is needed to determine the risk of denosumab-induced hypocalcemia for various bariatric surgical techniques, as well as to determine a safe and cost-effective way to select patients for extended calcium monitoring after denosumab administration.

This case demonstrates that the risk of profound hypocalcemia should be weighed when considering initiation of denosumab in a patient with a history of bariatric surgery. While bariatric surgery has been shown to decrease bone mineral density, a 2012 study in the British Medical Journal showed that bariatric surgery does not have a significant effect on fracture risk, at least over the short term [21]. So, perhaps, treatment of osteoporosis secondary to bariatric surgery can be less aggressive than treatment of primary osteoporosis. For those patients with a bariatric surgery history who are started on denosumab for osteoporosis, monitoring of calcium, phosphorus, and magnesium should be extended beyond the 14-day interval recommended in the Prolia® insert, and higher doses of calcium, phosphorus, magnesium, and vitamin D should be considered than those recommended for general denosumab use. Finally, patients should be educated about the importance of long-term adherence to supplementation, as well as what symptoms should prompt them to visit the emergency department.

## Data Availability

No data were used to support this study.

## Supplementary Materials

Supplementary Materials Appendix 1: CARE checklist.
