# Supplementary material for: Denosumab-Induced Hypocalcemia after Billroth II Gastric Bypass Surgery
Source: Case Rep Endocrinol. 2020 Jul 23;2020:8833723. doi: 10.1155/2020/8833723 (PMC7396013; doi:10.1155/2020/8833723)
Supplement: Supplementary Materials — Appendix 1: CARE checklist. [file 8833723.f1.pdf]

CARE Checklist of information to include when writing a case report

CARE case report guidelines

(cc) BY-NC-ND

Topic	Item	Checklist item description	Reported on Line
Title	1	The diagnosis or intervention of primary focus followed by the words "case report"	2
Key Words	2	2 to 5 key words that identify diagnoses or interventions in this case report, including "case report"	5
Abstract (no references)	3a	Introduction: What is unique about this case and what does it add to the scientific literature?	36-41
	3b	Main symptoms and/or important clinical findings	31
	3c	The main diagnoses, therapeutic interventions, and outcomes	32-34
	3d	Conclusion—What is the main "take-away" lesson(s) from this case?	41-45
Introduction	4	One or two paragraphs summarizing why this case is unique (**may include references**)	49-68
Patient Information	5a	De-identified patient specific information.	72-78
	5b	Primary concerns and symptoms of the patient.	78-84
	5c	Medical, family, and psycho-social history including relevant genetic information	72-76
	5d	Relevant past interventions with outcomes	N/A
Clinical Findings	6	Describe significant physical examination (PE) and important clinical findings.	86-93
Timeline	7	Historical and current information from this episode of care organized as a timeline	N/A
Diagnostic Assessment	8a	Diagnostic testing (such as PE, laboratory testing, imaging, surveys). Text	95-102
	8b	Diagnostic challenges (such as access to testing, financial, or cultural)	N/A
	8c	Diagnosis (including other diagnoses considered)	124-127
	8d	Prognosis (such as staging in oncology) where applicable	N/A
Therapeutic Intervention	9a	Types of therapeutic intervention (such as pharmacologic, surgical, preventive, self-care)	104-111
	9b	Administration of therapeutic intervention (such as dosage, strength, duration)	104-111
	9c	Changes in therapeutic intervention (with rationale)	N/A
Follow-up and Outcomes	10a	Clinician and patient-assessed outcomes (if available)	106-109
	10b	Important follow-up diagnostic and other test results	111-113
	10c	Intervention adherence and tolerability (How was this assessed?)	111-113
	10d	Adverse and unanticipated events	N/A
Discussion	11a	A scientific discussion of the strengths AND limitations associated with this case report	171-180
	11b	Discussion of the relevant medical literature **with references**.	117-169
	11c	The scientific rationale for any conclusions (including assessment of possible causes)	122-160
	11d	The primary "take-away" lessons of this case report (without references) in a one paragraph conclusion	182-193
Patient Perspective	12	The patient should share their perspective in one to two paragraphs on the treatment(s) they received	N/A
Informed Consent	13	Did the patient give informed consent? Please provide if requested	Yes ☐ No ☑
